# Animal and plant‐based proteins have different postprandial effects on energy expenditure, glycemia, insulinemia, and lipemia: A review of controlled clinical trials

**DOI:** 10.1002/fsn3.3417

**Published:** 2023-05-22

**Authors:** Zahra Dehnavi, Hanieh Barghchi, Ali Jafarzadeh Esfehani, Mehdi Barati, Zahra Khorasanchi, Farima Farsi, Andisheh Norouzian Ostad, Golnaz Ranjbar, Reza Rezvani, Mitra Rezaie Gorgani, Mohammad Safarian

**Affiliations:** ^1^ Department of Nutrition, School of Medicine Mashhad University of Medical Sciences Mashhad Iran; ^2^ Student Research Committee, Faculty of Medicine Mashhad University of Medical Sciences Mashhad Iran; ^3^ Metabolic Syndrome Research Centre Mashhad University of Medical Sciences Mashhad Iran; ^4^ Department of Pathobiology and Laboratory Sciences North Khorasan University of Medical Sciences Bojnurd Iran; ^5^ School of Medicine Mashhad University of Medical Sciences (MUMS) Mashhad Iran

**Keywords:** animal proteins, carbohydrate metabolism, energy metabolism, lipid metabolism, plant proteins, postprandial period

## Abstract

Dietary proteins have been shown to stimulate thermogenesis, increase satiety, and improve insulin sensitivity in the short and long term. Animal‐based proteins (AP) and plant‐based proteins (PP) have different amino acid profiles, bioavailability, and digestibility, so it seems to have various short‐ and long‐term effects on metabolic responses. This review aimed to compare the findings of controlled clinical trials on postprandial effects of dietary Aps versus PPs on energy expenditure (EE), lipemia, glycemia, and insulinemia. Data are inconclusive regarding the postprandial effects of APs and PPs. However, there is some evidence indicating that APs increase postprandial EE, DIT, and SO more than PPs. With lipemia and glycemia, most studies showed that APs reduce or delay postprandial glycemia and lipemia and increase insulinemia more than PPs. The difference in amino acid composition, digestion and absorption rate, and gastric emptying rate between APs and PPs explains this difference.

## Graphical abstract

Animal protein and plant protein may exert different postprandial effects that may be helpful in designing weight control programs, prevention, and treatment of chronic diseases.

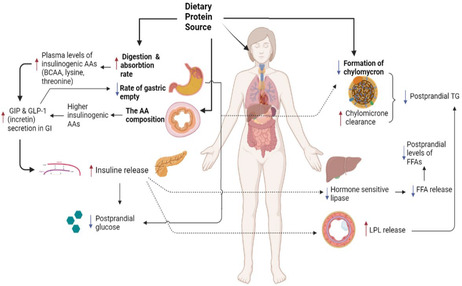

## 1 INTRODUCTION

Dietary proteins have been shown to exhibit beneficial effects on metabolic responses in the short and long term (Baba et al., 1999; Clifton et al., 2008), including increased thermogenesis, decreased energy intake, and improved insulin sensitivity (Gannon et al., 2003; Halton & Hu, 2004). However, regardless of the amount of dietary protein, not all protein sources seem to have the same metabolic effects because of differences in profile characteristics.

Animal‐based proteins (AP) and plant‐based proteins (PP) are different in terms of amino acid profile, bioavailability, and digestibility (Sá et al., 2020). Plant‐based proteins are low in some essential amino acids, such as lysine, leucine, and methionine (van Vliet et al., 2015; WHO, 2007). Therefore, PPs may have lower quality compared with APs. PPs also have lower digestibility (75%–80%) than APs (90%–95%). Additionally, plant proteins have lower enzyme accessibility due to their seed coats and rigid cell walls (Annor et al., 2017; Habiba, 2002).

The postprandial effects of APs and PPs on different metabolism markers have been previously studied (Acheson et al., 2011; Crowder et al., 2016; Veldhorst et al., 2009). It has been shown that APs induce more energy expenditure compared with PPs, which may be due to the higher thermogenic effect of some essential amino acids in APs compared with the amino acid content of PPs (Mikkelsen et al., 2000).

The various effects of APs and PPs on postprandial lipid and carbohydrate metabolism are possibly due to their different insulinotropic effects (Nilsson et al., 2004). Insulin release is differently affected by various amino acids. For instance, branched‐chain amino acids (BCAAs), including valine, leucine, and isoleucine, are known as insulinogenic amino acids and can induce insulin release in the short and long term (von Post‐Skagegård et al., 2006). APs and PPs differently affect the serum levels of incretins, including Glucose‐dependent insulinotropic polypeptide (GIP) and Glucagon‐like Polypeptide‐1 (GLP‐1), which affect insulin release and some enzymes involved in lipid metabolism, including lipoprotein lipase (LPL) and hormone‐sensitive lipase (Oliveira et al., 2011).

This review investigated the existing literature from controlled clinical trials to compare the postprandial effects of dietary protein sources (AP and PP) on expended energy (EE), lipemia, glycemia, and insulinemia. Furthermore, we aimed to clarify the possible potential mechanisms underlying the postprandial effects of different protein sources.

### 1.1 Postprandial energy expenditure

Total energy expenditure (TEE) is comprised of basal metabolic rate (BMR; 60%–80%), diet‐induced thermogenesis (DIT; 10%), and expended energy during physical activity (15%–30%) (Wang et al., 2001). The thermogenic effect of protein is the difference between the metabolizable energy value and gross energy value (Westerterp‐Plantenga et al., 2012). Protein has a higher thermogenic effect compared with other macronutrients.

The possible biological mechanism for the high thermogenic effect of proteins is the lack of storage capacity for proteins in the body to cope with high‐protein intakes, which lead to protein metabolization and thus increase thermogenesis (Gurr et al., 1980; Rothwell & Stock, 1987). The high thermogenic effect of protein may be attributed to (a) high Adenosine triphosphate (ATP) costs of peptide bonds (Garlick et al., 1991; Giordano & Castellino, 1997; Golden et al., 1977; Rennie et al., 1982), (b) the high‐energy cost of the pathways that involve protein metabolism, including gluconeogenesis and urea production (Stryer, 1995), and (c) increased proton‐pump activity in liver cell membrane following high‐protein meal ingestion (Forslund et al., 1999).

The protein synthetic response largely depends on the availability of essential amino acids, especially; leucine (Atherton et al., 2010; Volpi et al., 2003). After ingestion of APs, protein synthesis increases more than PPs, which is due to the increase in plasma essential AAs (e.g., leucine; Gorissen et al., 2016, 2017; Robinson et al., 2013; Yang et al., 2012).

Since APs and PPs have different amino acid compositions and different effects on protein synthesis, they may affect EE, DIT, and substrate oxidation (SO) differently in the postprandial period.

The findings of previous studies regarding the postprandial effects of APs and PPs on different markers of energy metabolism have been controversial (Table 1).

**TABLE 1:** Controlled clinical trials on the acute effects of protein source (AP vs. PP) on EE.

Reference	Study population	Study design	Test meals	Outcomes			
				EE	BMR	SO	DIT
Per B Mikkelsen et al. (2000) (Denmark; Mikkelsen et al., 2000)	22 young, overweight‐to moderately obese	Randomized, single‐blind, crossover, 3‐treatment trial design	Diets: Low‐fat, high pork‐meat protein diet (pork diet) Low‐fat, high‐soy‐protein diet (soy diet) Low‐fat, high‐carbohydrate diet (CHO diet)	Pork diet > soy diet (1.9%; *p* = .05) Pork diet > CHO diet (3.9%; *p* < .0001) Soy diet > CHO diet (1.9%; *p* < .05)	Pork diet > CHO diet (4.5%) Pork and soy diets (not significantly different)	‐	Pork diet > CHO diet (5.5%) Soy diet > CHO diet (1.9%) Pork and soy diets (not significantly different)
Kevin J Acheson et al. (2011) (Switzerland; Acheson et al., 2011)	23 lean, healthy subjects	Randomized, double‐blind, crossover, 5‐treatment trial design	High‐protein test meals: WPI Micellar CP SPI Carbohydrate test meal	WPI > micellar CP (*p* = .003) WPI > SPI (*p* < 0.0001) Micellar CP and SPI (not significantly different; *p* = .80) All of the pro test meals > CHO meal (*p* < .0001)	‐	Fat oxidation: WPI > SPI (*p* = .098) WPI > CHO (*p* < .0001) Protein oxidation: not significantly different between the 3 test meals	WPI > Micellar CP (*p* = .002) WPI > SPI (*p* = .001) All of the protein test meals > CHO meal (*p* < .0001)
Sze‐Yen Tan et al. (2010) (Australia; Tan et al., 2010)	12 healthy participants	Randomized, crossover, 3‐treatment trial design	Diets: Meat (lean beef and ham) Dairy (low‐fat milk, cheese, and yoghurt) Soy (as a plant alternative option)	Meat > soy (1.1%, but not significant)	‐	CHO oxidation: Not significantly different between the 3 test meals. Fat oxidation: Not significantly different among the 3 test meals. Pro oxidation: Meat (34.9 ± 11.1) < soy (39.5 ± 10.7) (*p* = .012)	‐
Aubree L Hawley et al. (2020) (USA) (Hawley et al., 2020)	15 young men 18–29 year and 15 old men 60–85 year of age	Randomized, single‐blind, crossover, 2‐treatment trial design	Beverages: Animal‐based protein test beverage (Chocolate WPI) Plant‐based protein test beverage (Chocolate pea protein isolate (PPI))	Not significantly different between WPI and PPI	‐	Fat oxidation: Not significantly different between WPI and PPI	Not significantly different between WPI and PPI
Caroline E. Melson et al. (2019) (North Carolina, United States) (Melson et al., 2019)	17 healthy adults	Randomized, double‐blind, crossover, 3‐treatment trial design	Breakfast smoothies: Plant‐based protein smoothie (whey concentrate and isolate) Animal‐based protein smoothie (soy isolate) Carbohydrate (CHO)	‐	‐	RER: Whey < CHO (*p* = .007) Soy < CHO (*p* = .015) WP and SP (not significantly different; *p* = .364)	Whey > CHO (*p* < .001) Soy > CHO (*p* < .001) Whey and Soy (not significantly different; *p* = .308)
Rita de Cassia Gonçalves Alfenas et al. (2010) (Brazil) (Alfenas et al., 2010)	24 subjects, aged 23.5 ± 3.95 years	Randomized, crossover, four 7‐day experimental sessions design	Shakes: CP SP WP No test proteins (control session)	‐	‐	RQ: Whey < Control (on days 1 and 7) (*p* ≤ .030) WP < SP (on days 1 and 7) (*p* ≤ .027)	SP > control (on days 1 and 7) (*p* ≤ .035) SP > WP (on day 7) (*p* = .024)

Abbreviations: <, Less than; >, More than; BMR, Basal Metabolic Rate; CHO, Carbohydrate; CP, Casein Protein; DIT, Diet‐Induced Thermogenesis; EE, Energy Expenditure; RER, Respiratory Exchange Ratio; RQ, Respiratory Quotient; SO, Substrate Oxidation; SP, Soy Protein; WP, Whey Protein; WPI, Whey Protein Isolate.

Some studies showed that APs increased postprandial EE and SO compared with PPs (Acheson et al., 2011; Mikkelsen et al., 2000; Tan et al., 2010). The higher EE and DIT due to AP ingestion may be due to the higher thermogenic response produced by a high biological value protein, consisting of a well‐balanced amino acid mixture, than a lower biological value protein (soy; Nielsen et al., 1994; Pitkänen et al., 1994). Moreover, leucine, which is found in larger amounts in animal proteins, has the most thermogenic effect (Tsujinaka et al., 1996). APs also have lower protein oxidation than PPs, suggesting that APs, like meat, can produce a protein‐sparing effect and might maintain lean body mass (Tan et al., 2010). On the other hand, some studies showed no significant difference in postprandial effects on EE, carbohydrate, and fat oxidation neither between APs and PPs nor between different AP proteins (Hawley et al., 2020; Melson et al., 2019).

In summary, although some studies declare that APs increase EE and SO more than PPs, the findings of controlled clinical trials are inconclusive, and there is a need for further studies to evaluate the effects of protein sources on postprandial EE and SO.

### 1.2 Postprandial glycemia and insulinemia

The quality and quantity of dietary protein could affect glycemic response. Dietary proteins induce insulin secretion and influence glycemic response, both in the long and short term (Bowen et al., 2007; Layman et al., 2003; von Post‐Skagegård et al., 2006). Therefore, high‐protein meals with low or moderate carbohydrate content increase insulin secretion due to the synergistic effect of high‐protein and low‐carbohydrate intake on insulin sensitivity and glucose uptake (Frid et al., 2005; Gannon et al., 2003).

**FIGURE 1:**
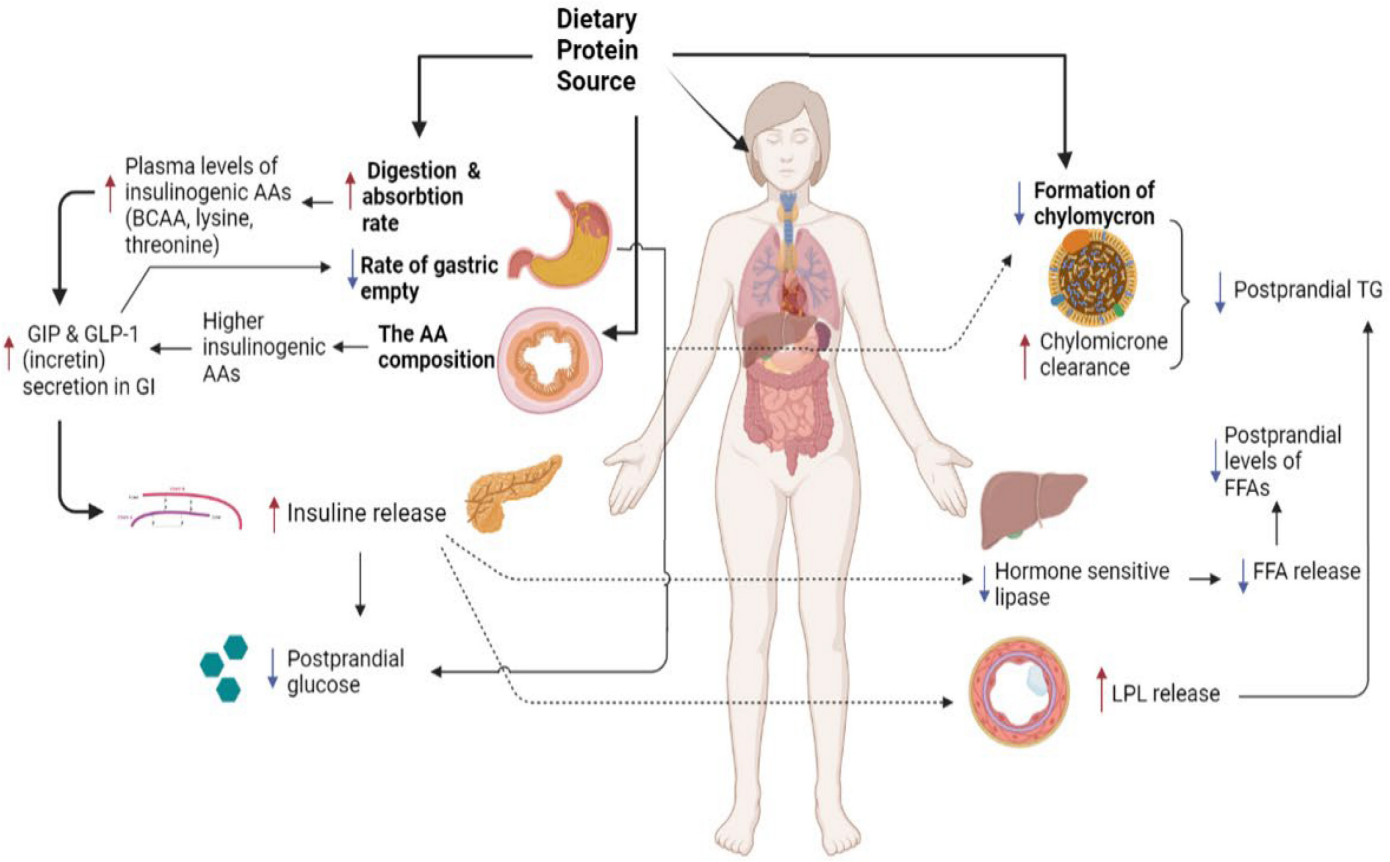
Schematic representation of postprandial glycemia, insulinemia, and lipemia modulated by dietary protein source.

Animal‐based proteins and PPs seem to affect insulin secretion and glucose uptake differently in the postprandial period (Frid et al., 2005). Some AAs, including BCAAs (valine, leucine, isoleucine), are known as insulinogenic amino acids and can increase insulin secretion in the short and long term (Schmid et al., 1992; von Post‐Skagegård et al., 2006). On the other hand, the digestion and absorption rate of AP and PP is different, which alters the serum levels of the gastric inhibitory polypeptide as an insulinotropic peptide (Jakubowicz & Froy, 2013). The faster the digestion of proteins, the faster the release of bioactive amino acids in the bloodstream and the higher stimulation of incretins secretion (Oliveira et al., 2011). Incretins, including Glucose‐dependent insulinotropic polypeptide (GIP) and Glucagon‐like Polypeptide‐1 (GLP‐1) stimulate insulin release and inhibit glucagon hormone secretion (Calbet & MacLean, 2002; Chacra, 2006; Johnston & Buller, 2005). The absorption and digestion rate of whey protein is faster than PPs. Whey protein contains higher concentrations of valine, leucine, isoleucine, and lysine, which have insulinotropic effects than PP. Therefore, whey protein can induce insulin secretion and improve insulin sensitivity and reduce glycemic response to a greater extent compared with PPs (Akhavan et al., 2010; Pal & Ellis, 2010; van Loon et al., 2000). In addition to digestion and absorption rate, the different gastric emptying rates may explain the differences in the glycemic response of AP and PPs (Lang et al., 1999). The concept of fast and slow proteins was first introduced by Boirie et al. (1997) (Boirie et al., 1997); hence, slow proteins reduce and delay glycemic responses due to gastric emptying (Figure 1).

Table 2 summarizes controlled clinical trials, which evaluated the effects of different protein sources (AP vs. PP) on postprandial glycemia and insulinemia.

**TABLE 2:** Controlled clinical trials on the acute effects of protein source (AP vs. PP) on glycemia and insulinemia.

Reference	Study population	Study design	Test meals	Outcomes	
				Glycemia	Insulinemia
Ann Bjørnshave et al. (2019) (Denmark) (Bjørnshave et al., 2019)	28 subjects above 18 years with the Metabolic syndrome	Randomized, crossover design	Fat‐rich meal Pre‐meals (15 min before the fat‐rich meal) with three different protein types: Whey (−15 and −30 min) Casein (−15 min) Gluten (−15 min)	The glucose responses fluctuated between 5.1 and 6.3 mmoL/L at 0–120 min after 4 meals. No effect of protein quality (*p* = .93)	Ins after all 4 pre‐meals ↑ Ins after 15 min: WP (−15 min) > gluten Casein > gluten (*p* = .0062) Max Ins: WP (−15 min), WP (−30 min) and casein: after 15 min Gluten: after 30 min
Anestis Dougkas et al. (2018) (Sweden) (Dougkas & Östman, 2018)	28 healthy adult men	Randomized SB, crossover design	Rice puddings: Animal‐based protein (milk proteins) (AP) A blend of plant proteins (oat, pea and potato) (VP) Mixture of the two (50:50) (MP) Carbohydrate‐rich meal (CHO)	Glucose concentrations: CHO > Protein‐rich meals AP < VP No differences between MP and VP or AP meals Significant difference between all groups (*p* = .001)	Insulin concentrations: AP > VP (but did not reach statistical significance) Not significantly different between all groups (*p* = .06)
Christina M. Crowder et al. (2016) (USA) (Crowder et al., 2016)	12 Normal weight and 8 overweight women	Randomized, crossover design	Commercially breakfast sandwiches: Plant‐based protein (PP) Animal‐based protein (AP)	Postprandial blood glucose (at 30 min): PP (126.8 ± 4.4 mg/dL) > AP (112.1 ± 3.9 mg/dL) (*p* < .05) Percent change in blood glucose response (from the postprandial peak at 30 min to 120 min): AP (−26.9 ± 4.3%) < PP (−46.5 ± 4.9%) (*p* < .01)	‐
Winder Tadeu Silva Ton et al. (2014) (Brazil) ( Silva Ton et al., 2014)	10 subjects with normal body weight and fasting glucose	Randomized, crossover design	Protein shakes: WP SP Egg white Control drink (spring water + calories‐free blackberry powder juice) Glucose solution (anhydrous glucose)	Postprandial Glycemia: After 15 min: WP < SP and egg white (*p* = .007) After 30 min: WP < SP and egg white (*p* = 0.001) After 45 min: WP < egg white (*p* ≤ 0.02) After 60 min: Not significantly different between 3 protein drinks (*p* > .05)	‐
Jens Holmer‐Jensena et al. (2013) (Denmark) (Holmer‐Jensen et al., 2013)	11 obese non‐diabetic subjects	Randomized, crossover design	Soups with 45 gr protein of: Cod protein (Cod‐meal) The spray dried WPI (Whey‐meal) Gluten (Glu‐meal) CP (Cas‐meal)	Glucose net iAUC (0 to 120 min): A statistically significant main effect of treatment group (*p* = .0022) The glucose increment after Whey‐meal < other meals	Insulin net iAUC (0 to 120 min): A statistically significant main effect of treatment group (*p* = .0001) The initial insulin response: Whey‐meal > Cod‐meal (67%) Whey‐meal > Glu‐meal (88%) Whey‐meal > Cas‐meal (47%)
Kevin J Acheson et al. (2011) (Switzerland) (Acheson et al., 2011)	23 lean, healthy subjects	Randomized, DB, crossover design	High‐protein test meals: WPI Micellar casein SPI CHO test meal	↓ peak glycemia: Both after animal and plant‐based proteins Glycemia IAUC at 120 min and end test: protein meals < CHO meal (*p* < .0001) Not significantly different between 3 protein meals (*p* > .05) Glycemic index after protein meals: WP = 33 ± 3% CP = 36 ± 3% SP = 32 ± 4% (*p* < .01)	Insulinemia IAUC at 120 min: WP > glucose (*p* = .02) WP > SP (51%; *p* = .03) WP > CP (43%; *p* = .07) Insulinemia IAUC end test: WP > Glucose (*p* < .01) WP > SP (*p* = .08)
Lene S Mortensen et al. (2009) (Denmark) (Mortensen et al., 2009)	12 diabetic subjects	Randomized, crossover design	Soups with 45 gr protein of: Casein protein (CP) Whey protein (WP) Cod protein (Cod‐P) Gluten protein (GP)	The glucose iAUC: WP < other meals (at both 360 min (*p* = .015) and 480 min (*p* = .015))	The insulin responses: No significant differences between the 4 test meals
Mikael Nilsson et al. (2004) (Denmark) (Nilsson et al., 2004)	12 healthy subjects	Randomized, crossover design	Reconstituted milk Cheese Whey Cod Wheat gluten (low (GL) & high (GH)) Anequi CHO load of white‐wheat bread (reference meal)	Glucose AUC (0–90 min): Milk (−57%) & whey (−62%) < reference meal No significant differences between the reference and the GL, GH, cod, and cheese meals. BG response at 30 min: Milk and whey > cod (*p* < .05) GH & GL > whey (*p* < .05)	Insulin AUC (0–90 min): Whey > all other meals (*p* < .05) Milk & cheese > GL‐meal (*p* < .05)
V Lang et al. (1999) (France) (Lang et al., 1999)	9 healthy normal‐weight men	Randomized, crossover design	High‐calorie (3.6 MJ) and low‐calorie (1.8 MJ) Test lunches with 23% energy as protein: CA GE Soy	PG: Significantly fluctuated across the postprandial period after all meals (*p* < .001) Time × protein source interaction for glucose (*p* < .005) was observed No protein source effect was found for plasma glucose and glucose AUCs	Time × protein source interaction for insulin (*p* < .001) was observed Insulin AUCs (following 3.6 MJ lunches): GE‐high < Soy protein‐high (*p* < .05) Insulin AUCs (following 1.8 MJ lunches): GE‐low < CA‐low (*p* < .05)

Abbreviations: <, Less than; >, More than; ↑, Increase; ↓, Decrease; BG, Blood Glucose; CHO, Carbohydrate; CP, Casein Protein; DB, Double‐blinded; GE, Gelatin; iAUC, Incremental Area Under The Curve; PG, Plasma Glucose; SB, Single‐blinded; SPI, Soy protein isolate; WPI, Whey protein isolate.

The findings of most previous studies were in favor of the postprandial glucose‐lowering effects of APs compared with PPs (Bjørnshave et al., 2019; Crowder et al., 2016; Dougkas & Östman, 2018; Holmer‐Jensen et al., 2013; Silva Ton et al., 2014).

Silva Ton et al. (2014) compared the postprandial effects of three types of proteins, including whey, egg white, and soy proteins, on glycemia among 10 healthy normoglycemic subjects. They concluded that whey protein resulted in a lower glycemic response than soy and egg white proteins at 15, 30, and 45 min after the meal. Furthermore, postprandial glycemia was more stable after whey protein consumption.

The absorption and digestion rate of whey protein is faster than PP. Whey protein contains higher concentrations of valine, leucine, isoleucine, and lysine, which have insulinotropic effects than PP. Therefore, whey protein can induce insulin secretion and improve insulin sensitivity, contributing to a lower glycemic response than PP (Akhavan et al., 2010; Pal & Ellis, 2010; van Loon et al., 2000). Hence, whey protein exerts a more significant effect on glycemic response.

Dougkas and Östman (2018) compared the postprandial effects of breakfast meals containing PP (a blend of oat, pea, and potato), AP (milk), and a 50:50 mixture. The results showed that plasma glucose was higher after consuming the PP compared with the AP, possibly due to the higher insulin levels after ingestion of the AP meal. However, this difference was not statistically significant.

In another randomized, crossover study by Crowder et al. (2016), the effects of AP versus PP on postprandial metabolic response, including postprandial glycemia, were compared (Crowder et al., 2016). The authors showed that postprandial glucose was higher after PP meal ingestion than after AP meals. Moreover, the percent change in glucose response from the postprandial peak was lower following the AP meal ingestion than a PP meal.

In summary, APs, especially whey protein, result in lower glycemic and higher insulin responses than PPs, including soy or peas. The amino acid composition, digestion and absorption rate, and gastric emptying rate between different protein sources may explain these differences.

### 1.3 Postprandial lipemia

Postprandial lipemia is associated with meal composition, and it is demonstrated that the amount and type of food macronutrients can affect the duration and increment of postprandial lipemia (Bozzetto et al., 2020; Draper et al., 2019; Dubois et al., 1998; Jeppesen et al., 1995; Miyoshi et al., 2014; Thomsen et al., 1999).

Protein quantity and quality may also affect postprandial lipemia. It has been proven that consuming a high‐protein diet enriched with AP leads to lower postprandial chylomicronemia than a low‐protein diet (25% and 14%, respectively; Mamo et al., 2005). APs and PPs have different effects on postprandial lipemia. The mechanism of the different effects of APs and PPs on postprandial lipemia is not clearly understood. However, some hypotheses have been proposed for these findings. One mechanism might be related to the formation and clearance of chylomicron (Mortensen et al., 2009). Another mechanism might be the different effects of protein sources on lipoprotein lipase (LPL) release (Acheson et al., 2011; Eckel, 1989). Lipoprotein lipase is the essential enzyme in regulating the metabolism of lipids and lipoproteins; it plays a vital role in the hydrolysis of the TG content of these lipoproteins (Eckel, 1989; Kersten, 2014). Another reason might be the difference in the insulinogenic amino acid content of different protein sources that results in different insulin stimulation (Acheson et al., 2011; Nilsson et al., 2004). Insulin is an essential stimulant for LPL and hormone‐sensitive lipase, affecting TG levels and postprandial lipemia (Draper et al., 2019; Holmer‐Jensen et al., 2013). Another mechanism might be the different effects of protein sources on incretins (GIP and GLP‐1) release that results in different gastric emptying and insulinogenic effects (Bjørnshave et al., 2019). The other possible mechanism for this observation is the difference in protein precipitation rate, which affects gastrointestinal transit (Stanstrup et al., 2014; Figure 1).

Table 3 summarizes the findings of controlled clinical trials on the effects of APs and PPs on postprandial lipemia.

**TABLE 3:** Controlled clinical trials on the acute effects of protein source (AP vs. PP) on lipemia.

Reference	Study population	Study design	Test meals	Outcomes	
				TG	FFA
Ann Bjørnshave et al. (2019) (Denmark) (Bjørnshave et al., 2019)	28 subjects >18 years with the MS	Randomized, crossover design	Fat‐rich meal Pre‐meals (15 min before the fat‐rich meal) with 3 different protein types: WP (−15 and −30 min) CP (−15 min) Gluten (−15 min)	The postprandial TG Response: No effect of the type of protein pre‐meal (*p* = .95) and its timing (*p* = .87)	Postprandial NEFA suppressions: Similar after all 4 groups; Reached a minimum at 60 min and returned to fasting levels at 360 min. No effect of type of protein pre‐meal (*p* = .79) or timing (*p* = .22)
Jan Stanstrup et al. (2014) (Denmark) (Stanstrup et al., 2014)	11 Obese, non‐diabetic subjects	Randomized, SB, crossover design	Soups with 45 gr protein of: WPI Calcium caseinate Gluten Cod protein	‐	MCFA, including α Hydroxydecanoic, lauric and myristic acid levels after: WI meal < other meals (at one or more time points after 2 h) The WI meal ⇒ ↓ levels of a number of FA The GLU meal ⇒ ↑ levels of a number of unidentified hydroxy and dicarboxylic FA
Jens Holmer‐Jensena et al. (2013) (Denmark) (Holmer‐Jensen et al., 2013)	11 obese non‐diabetic subjects	Randomized, crossover design	Soups with 45 gr protein of: Cod protein (Cod‐meal) The spray dried WPI (Whey‐meal) Gluten (Glu‐meal) CP (Cas‐meal)	Plasma TG: Significant (*p* = .048) main effect of treatment group for net iAUC (0–360 min) Cod‐meal > Whey‐meal (61%) Glu‐meal > Whey‐meal (66%) Supernatant TG: Significant (*p* = .03) main effect of treatment group for net iAUC (0–360 min) Cod‐meal > Whey‐meal (73%) Glu‐meal > Whey‐meal (61%)	Significant (*p* = .0092) main effect of treatment group for NEFA net iAUC (0–360 min) Suppression of NEFA: Whey‐meal and Cas‐meal > Cod‐meal and Glu‐meal (*p* < .05)
Kevin J Acheson et al. (2011) (Switzerland) (Acheson et al., 2011)	23 lean, healthy subjects	Randomized, DB, crossover, 5‐treatment trial design	High‐protein test meals: WPI Micellar CP SPI CHO test meal	Peak concentrations of TG at 240 min: Soy: 1.09 ± 0.12 mmol/L (*p* = .003) Whey: 1.05 ± 0.12 mmol/L (*p* = .017) Casein: 0.99 ± 0.10 mmol/L (*p* = .003) (*p*‐value not reported) Time of TG response to test meal: −45 min after the high‐CHO meal ‐Took longer to respond to the protein‐containing test meals	↓ FFA (*p*, .0001) after each test meal, and continued for a further 120–180 min. ↑ FFA at 240 min, in all tests FFA concentrations by the end of the test: Whey > CHO > Casein > Soy (*p*‐value not reported)
Lene S Mortensen et al. (2009) (Denmark) (Mortensen et al., 2009)	12 diabetic subjects	Randomized, crossover design	Soups with 45 gr protein of: CP 2.WP Cod‐P GP	TG concentrations of the chylomicron‐poor fraction: Not significantly different between the meals. The iAUC of TG in plasma (at 360 min): WP < 3 other meals (*p* = .008) The iAUC of TG in the chylomicron‐rich Fraction (at 360 min): WP < 3 other meals (*p* = .004) WP ⇒ delayed rise in p‐TG and in the chylomicron‐rich fraction, with a peak appearing 3 h later than with the 3 other meals	FFA concentrations (at 240 min): WP < other 3 meals (*p* < .05) Total AUC for FFA responses (at 360 min): WP < other 3 meals (*p* < .001) Total AUC for FFA responses (at 480 min): WP < Cod‐P (*p* = .011) Not differ from the response to the GP and CP

Abbreviations: <, Less than; >, More than; ↑, Increase; ↓, Decrease; CHO, Carbohydrate; DB, Double‐blinded; FFA, Free Fatty Acid; GP, Gluten Protein; iAUC, Incremental Area Under The Curve; MCFA, Medium Chain Fatty Acid; MS, Metabolic Syndrome; NEFA, Non‐Esterified Fatty Acid; SB, Single‐blinded; SPI, Soy protein isolate; TG, Triglyceride; WPI, Whey protein isolate.

Previous studies have reported that APs could ameliorate postprandial lipemia by reducing TG levels or free fatty acids (FFAs) compared with PPs (Holmer‐Jensen et al., 2013; Mortensen et al., 2009). Some studies also showed that whey protein was associated with lower postprandial serum lipids and reduced TG content of chylomicron‐rich supernatant (Acheson et al., 2011; Holmer‐Jensen et al., 2013; Mortensen et al., 2009).

In contrast, Bjørnshave et al. (2019) reported no significant relationship between the type of ingested protein and postprandial lipemia (Bjørnshave et al., 2019).

The possible mechanisms for the greater effect on postprandial lipemia observed after whey protein ingestion might be as follow; whey protein induces insulin release, increases the activity of LPL, and leads to a lower postprandial lipemia compared with other proteins. On the other hand, increased insulin levels after consuming whey protein inhibits hormone‐sensitive lipase and suppresses FFA release from adipose tissue.

Furthermore, some studies have mentioned that whey protein delays gastric emptying compared with casein (Acheson et al., 2011; Hall et al., 2003). Delayed gastric emptying may result in delayed and even slower postprandial peak levels of TG. However, the results of some previous studies regarding the difference in gastric transition were in contrast to the findings of the mentioned studies (Calbet & Holst, 2004; Mortensen et al., 2009). A possible reason for this controversy might be the difference in protein precipitation rates. For instance, different precipitation rates of casein compared with whey protein may result in different liquid and solid phases, where the former has a faster transit. Another possible explanation is the various effects of protein sources on GLP‐1 that may reduce stomach emptying. However, some studies showed no difference in GLP‐1 responses after the intake of AP (whey, casein, or cod) or PP (gluten) in subjects with diabetes (Mortensen et al., 2009) or metabolic syndrome (Bjørnshave et al., 2019).

In summary, few studies have compared the differences in the effects of AP and PP on postprandial lipemia and indicated that APs, in particular whey protein, result in a lower and delayed postprandial plasma TG elevation in comparison to other protein sources. Nevertheless, the distinct effects and mechanisms of action of APs and PPs on postprandial lipemia have not yet been documented. Thus, there is a need for further studies to evaluate the effects of different protein sources on postprandial lipemia.

## 2 CONCLUSION

Based on the findings of this review of control clinical trials, APs and PPs may have different effects on postprandial metabolism and physiology, including EE, glycemia and lipemia. Data are inconclusive concerning the postprandial effects of APs and PPs. However, there is some evidence indicating that APs increase postprandial EE, DIT, and SO more than PPs, which may be due to the higher thermogenic response produced by a high biological value protein consisting of a well‐balanced amino acid mixture. APs also found to reduce or delay postprandial glycemia and lipemia and increase insulinemia more than PPs.

Knowing the differences in postprandial effects of APs and PPs may provide an opportunity for weight control programs and prevention and management of chronic diseases. However, further well‐designed acute phase and long‐term studies are needed to evaluate the exact metabolic differences between APs and PPs and to identify the underlying mechanisms for these effects.

## AUTHOR CONTRIBUTIONS

**Zahra Dehnavi:** Conceptualization (equal); writing – original draft (equal). **Hanieh Barghchi:** Writing – original draft (equal). **Ali Jafarzadeh Esfehani:** Data curation (equal). **Mehdi Barati:** Visualization (equal). **Zahra khorasanchi:** Writing – review and editing (equal). **Farima Farsi:** Data curation (equal); writing – review and editing (equal). **Andisheh Norouzian Ostad:** Writing – review and editing (equal). **Golnaz Ranjbar:** Writing – review and editing (equal). **Reza Rezvani:** Conceptualization (equal); supervision (equal). **Mitra Rezaie Gorgani:** Conceptualization (equal); supervision (equal). **Mohammad Safarian:** Conceptualization (equal); supervision (equal).

## CONFLICT OF INTEREST STATEMENT

The authors also declare that they have no conflict of interest.

## ETHICAL APPROVAL

No ethical approval was required, as this is a review article with no original research data.

## INFORMED CONSENT

There were no study participants in this review article, and informed consent was not required.

## Data Availability

The data that support the findings of this study are available on request from the corresponding author.
